# Three-Dimensional (3D) Printed Silver Nanoparticles/Alginate/Nanocrystalline Cellulose Hydrogels: Study of the Antimicrobial and Cytotoxicity Efficacy

**DOI:** 10.3390/nano10050844

**Published:** 2020-04-28

**Authors:** Carlo Bergonzi, Giulia Remaggi, Claudia Graiff, Laura Bergamonti, Marianna Potenza, Maria Cristina Ossiprandi, Ilaria Zanotti, Franco Bernini, Ruggero Bettini, Lisa Elviri

**Affiliations:** 1 Food and Drug Department, University of Parma, Parco Area delle Scienze 27/a, 43124 Parma, Italy; 2 Department of Chemistry, Life Sciences and Environmental Sustainability, University of Parma, Parco Area delle Scienze 17/A, 43124 Parma, Italy; 3 Department of Veterinary Science, University of Parma, Strada del Taglio 10, 43126 Parma, Italy

**Keywords:** 3D printing, alginate/nanocrystalline cellulose/silver nanoparticles hydrogels, antimicrobial activity, HepG2 cytotoxicity

## Abstract

Here, a formulation of silver nanoparticles (AgNPs) and two natural polymers such as alginate (ALG) and nanocrystalline cellulose (CNC) was developed for the 3D printing of scaffolds with large surface area, improved mechanical resistance and sustained capabilities to promote antimicrobial and cytotoxic effects. Mechanical resistance, water content, morphological characterization and silver distribution of the scaffolds were provided. As for applications, a comparable antimicrobial potency against *S. aureus* and *P. aeruginosa* was demonstrated by in vitro tests as function of the AgNP concentration in the scaffold (Minimal Inhibitory Concentration value: 10 mg/mL). By reusing the 3D system the antimicrobial efficacy was demonstrated over at least three applications. The cytotoxicity effects caused by administration of AgNPs to hepatocellular carcinoma (HepG2) cell culture through ALG and ALG/CNC scaffold were discussed as a function of time and dose. Finally, the liquid chromatography-mass spectrometry (LC-MS) technique was used for targeted analysis of pro-apoptotic initiation and executioner caspases, anti-apoptotic and proliferative proteins and the hepatocyte growth factor, and provided insights about molecular mechanisms involved in cell death induction.

## 1. Introduction

The development of innovative biomaterials as advanced therapeutic solutions is continuously under investigation. To reach this aim, nowadays, natural, biocompatible, biodegradable materials combined with nano-systems are largely developed. Among applications, innovative biomaterial-based devices suitable for the treatment of infected wounds without inducing antimicrobial resistance or with anti-tumoral properties are particularly attractive.

Bacterial wound infection in humans often represents the major cause of severe pathologies development and chronicization, rather than the main obstacle to repair of the soft tissues concerned [1,2,3,4,5]. Furthermore, several clinical observations showed that chronic inflammatory diseases are statistically correlated with cancer development as a consequence in different tissues and organs [6].

The typical initial management of such aggressive infections is enrolled by persistent and broad-spectrum antibiotic administration, that can sometime transform into a clinically relevant and potentially dangerous condition, due to the predisposition of the tissues to host resistant species (e.g., methicillin resistant *Staphylococcus aureus* (Gram^+^; aerobic)—MRSA). MRSA and resistant *Pseudomonas* (Gram^−^; anaerobic) spp., as well as several among bacteria inhabiting chronic ulcers, are particularly prone to produce biofilms, a sort of protective polymeric matrix that mainly acts as a physical barrier to the permeation and action of antimicrobial agents [7], making many traditional therapies ineffective.

Advanced medications can be considered a consistent synergic aid for the prevention and/or management of the situations described. In general, polymeric hydrogel scaffolds are retained as worthwhile for their biocompatibility, biodegradability and capacity to keep the wound bed moist to prevent tissue dehydration while absorbing excessive exudates [8]. However, naturally occurring saccharide polymers can result in being too easily and quickly biodegradable in living microenvironments rather than indeed presenting poor mechanical properties in terms of consistency and elasticity [9,10].

Calcium alginate (ALG) is worldwide considered a highly absorbent, biocompatible and biodegradable hydrogel suitable for dressing purposes [11], it derives from seaweed, is generally regarded as safe and its known strength relies on the capacity to keep the wound environment moist, favoring healing as well as the formation of granulation tissue and being easily removable with painless dressing changes [12,13].

Crystalline nanocellulose (CNC) is a promising biomaterial derived from different cellulose sources by means of various processing methodologies [14]. The cellulose nanoparticles are characterized by elevated surface area and crystallinity, they have a very large elasticity modulus, and they have high strength, low toxicity and high biocompatibility. For these reasons CNC is a good candidate to be considered in many fields, including biomedical applications and especially in the use of 3D printing for tissue-engineered scaffolds and wound healing [15,16,17]. CNC has been employed in hydrogel preparation for strengthening mechanical properties such as elasticity and resistance to breakage [18].

Antimicrobial substances, especially silver, were already included in calcium alginate pharmaceutical forms [19]. L. Inbathamizh et al. described the effectiveness of plant-derived silver nanoparticles (AgNP) in terms of antimicrobial activity and anticancer activity on the hepatocellular carcinoma cell line (HepG2) using concentrations around 1 mg/mL [20].

Furthermore, recently Zhang et al. reviewed the literature on the topic, focusing on the clarification of anticancer mechanisms of action of AgNP, individuated in activities such as induction of stress to the cell endoplasmic *reticulum* and production enhancement of reactive oxygen species, that brings a cascade of events that cause damages to organelles and DNA provoking cell death [21], highlighting the wide spectra of potential applications of AgNPs. A particular focus was dedicated to their potential employment as next-generation anticancer therapeutic agent, investigated by both academic and industrial research in order to circumvent adverse effects caused by chemo- and radiation-therapies [22], rather than fighting antibiotic resistance through a specific antimicrobial activity [23].

Incorporation of AgNPs in calcium alginate-based hydrogels reinforced with cellulose nanocrystals, to properly improve the mechanical resistance, might be advantageous in order to combine the pharmaceutical properties of the metal nanoparticles with the physical features provided by the hydrogel intended as a biocompatible drug-delivery platform. Moreover, their preparation by means of ad hoc developed processes can be tailored in order to tune their shape, size, structure, moisture, mechanical characteristics and thus overall efficiency. The most versatile technique, suitable to manage hydrogel materials, considered almost semi-solid for their high viscosity, was identified in a previously reported 3D printing method by Elviri et al. [24]. The technique permits the easy setting of loaded drug amounts and arbitrarily choosing the size/dimension parameters with high reproducibility [24], in order to obtain hydrogels characterized by very high surface area/volume ratio and to limit drug dosages [25].

Here, ALG and CNC were chosen as the main excipients for the production of robust and highly reproducible 3D printed medication prototypes embedding AgNPs; their characterization was performed from a physical/mechanical point of view. In vitro antimicrobial activity against *S. aureus* and *P. aeruginosa* and cytotoxicity toward HepG2 cells were assayed. HepG2 cells present epithelial morphology and grow in vitro adherent to the culture substrate. This cell line also shows the peculiarity of being highly differentiated both from a functional and morphological point of view, resulting in being very useful in investigations concerning toxicity, cellular trafficking at the molecular level, and drug targeting [26].

Finally, investigations on cytotoxicity effects caused by the administration of AgNPs to HepG2 cells through scaffold inoculation were proposed by the liquid chromatography-mass spectrometry technique, deepening what are the molecular mechanisms involved in cell death induction evaluating the expression of protein markers [27].

## 2. Materials and Methods

### 2.1. Materials

Sodium alginate (Ph.Eur. grade; molecular weight by gel filtration chromatography (GFC) 180–300 kDa; slowly soluble in water) was from Carlo Erba (Milan, Italy).

Tribasic sodium citrate dihydrate (Lot. 1986C100), urea and calcium chloride were from Carlo Erba. Trypsin, bovine serum albumin (BSA), dimethylsulphoxide, thyazolyl blue tetrazolium bromite (MTT), Tris-HCl, ethylene diammino tetraacetic acid (EDTA), DL-ditiothreitol (DTT), iodoacetamide (IAA), ammonium bicarbonate, trifluoroacetic acid (TFA), acetonitrile were from Sigma-Aldrich^®^ (Darmstadt, Germany). Dulbecco’s Modified Eagle’s Medium (DMEM) was from Gibco (Thermo Fisher Scientific, Waltham, MA, USA). Fetal Serum Bovine (FBS) and Dulbecco’s phosphate buffered saline (PBS) were from Euroclone (Milan, Italy).

AgNP suspension (nominal concentration: 1000 ppm) was prepared by AgNO_3_ reduction in water with NH_4_, stabilized with poly-vinyl-alcohol (PVA), [28]. Dynamic Light Scattering particle size characterization of the AgNP suspension (1000 ppm) indicated an average hydrodynamic radius of about 60–80 nm. The sol shows a moderate polydispersity (polydispersity index 0.4).

Protein assay dye reagent concentrate was from Bio-Rad. CNC suspension was obtained by acid hydrolysis of cotton linter following the procedure described by Basile et al. [29].

### 2.2. Methods

#### 2.2.1. Ink Preparation for 3D Printing

Ink formulations for 3D printing were developed following the same preparation principles and critical parameters used for scaffold production in previous research works (e.g., viscosity, polymer concentrations, homogeneity) [29,30,31].

In particular, ALG (5% *w/v*) alone or ALG (5% *w/v*) and CNC (3% *w/v*) powders were accurately weighted and homogeneously dispersed in an ultrapure water or in a aqueous suspension of AgNP (different concentrations). The blends were then kept under magnetic stirring at room temperature (22–24 °C) over night to let sodium alginate completely dissolve, physically embedding CNC and AgNP. Once ready to use, the formulations were stored at 4 °C in the dark for up to one week.

#### 2.2.2. Three-Dimensional (3D) Printing and Scaffold Production

A 3D printer built in-house was specifically designed for manipulation of aqueous viscous materials intended for hydrogel scaffold production. The surface plate is cooled at −14 °C, while the viscous material (ranging 8–40 k cP) [32], instantaneously solidifies during construction through layer by layer deposition. At the end of the printing procedure, the solid (frozen polymeric blend) structure was fixed (30 min in 3M CaCl_2_ solution) in its hydrogel form through the formation of the characteristic “egg box structure” [33]. Scaffolds were then washed with ultrapure water for the removal of calcium chloride excesses and stored at 4 °C.

#### 2.2.3. Scaffold Characterization

Water content was determined by gravimetric analysis: 5 scaffolds of each type were gently tamponed on filter paper and weighted, then placed in oven at 40 °C (till constant weight) and weighted again. The % water content was calculated using the formula:

100 − (100 × D_w_)/W_w_ (1)

where “D_w_” stands for “dry weight” and “W_w_” for “wet weight”.

The mechanical parameters evaluated were elasticity (Young’s modulus) and elongation % at break. The tests were performed by a tractional dynamometer (AG M1, Acquati, Milan, Italy); scaffold nominal sizes were: 20 layers × 5 cm × 1.5 cm; thicknesses were determined by a digital thickener (Mitutoyo, Japan) taking measurements from 6 distinct points along the scaffolds. Distance between clips was prefixed at ±25 mm, traction speed 25 mm/min, 5 DaN top head.

Force applied by the tensile tester (N) and net movement (mm) was continuously recorded and digitalized by PowerLab 400 board and Scope 3.5 software. Elongation at break (% strain) and Young’s modulus were calculated from the relevant stress–strain curves, taking into consideration the linear portion, corresponding to the elastic behavior of the specimens. In particular, Young’s modulus was calculated using the formula:

E = σ/ε(2)

where σ corresponds to stress (applied force/cross section area) and ε to strain (net elastic elongation). Elongation percentage was calculated as the ratio (100*ε)/specimen length. All analyses were conducted in triplicate for each type of sample.

#### 2.2.4. Elemental (Ag) Distribution within Scaffolds and Overall Morphology

The distribution of silver nanoparticles in the scaffold was evaluated by a scanning electron microscope (Jeol JSM 6400)(Jeol Spa, Milan, Italy) equipped with an Oxford Instruments Link Analytical Si (Li) energy-dispersive system detector (SEM-EDS). All the samples were carbon coated before the analysis. The data was processed by INCA built-in software.

Scaffolds were characterized as well in terms of macro/micro-porosity; 5-layer (1.6 cm × 1.6 cm) specimen were de-hydrated by washing in increasing percentages of ethanol, 10 min each, till absolute ethanol. Then, Critical Point Drying (Balserz Union, FL, USA) was employed (70 atm, 37 °C) to eliminate ethanol and obtain dried samples with an unaltered structure. Scaffolds were finally accurately cut/broken in order to obtain pieces exposing the cross sections, rather than surface area. The last step consisted in sample gold sputtering, producing a thin gold coating for optimal electrical conduction. Finally, after proper fixing on ad hoc stubs, scaffolds were observed through a Philips 501 SEM at magnifications ranging from 80× to 320×. Photographs were processed by means of ImageJ software (NIH, Bethesda, MD, USA), measuring the filaments patterned by the printer and collecting Feret’s diameters of 150 randomly taken micropores both of the surface and the cross section of the scaffolds.

#### 2.2.5. Antimicrobial Activity

Antimicrobial activity evaluation against two strains of bacteria (Gram^+^ and Gram^−^) was assessed. Multi-drug resistant strains of *Staphilococcus aureus* (ATCC 25923) (Manassas, VA, USA) and *Pseudomonas aeruginosa* (ATCC 27853)(Manassas, VA, USA) were considered for such test, since they are responsible of frequent infections in chronic wounds [34].

Diffusion disk method (or Kirby-Bauer technique) was adopted and *ad hoc*—modified in order to assay 3D printed hydrogel scaffolds [35]. 15 layered scaffolds (15 × 15 mm^2^) were produced using the developed formulation, ALG and ALG/CNC AgNP-free scaffolds, as control, and cut with circular 6 mm diameter stamps. After that, samples were sterilized in 70% *v/v* ethanol [29], rinsed and stored in sterile water at 4 °C until use. AgNPs were tested at different concentrations, ranging from 100 ppm to 1 ppm, in order to define the MIC.

Bacteria were seeded in pure culture and inoculated in a Mueller Hinton Broth at 37 °C under aerobic conditions for 1–2 h (0.5 McFarland). Bacterial suspension was seeded through the use of sterile tampons on a Mueller Hinton Agar terrain (carefully covering the entire Petri dish). Scaffolds were then applied by the use of sterile forceps. Each test was conducted in duplicate. A negative and positive control were established: a negative one with the scaffold only, to ensure it was not contaminated and a positive one consisting in a Petri dish seeded with bacteria but free of scaffolds. Finally, all the plates were incubated at 37 °C for 18–24 h. Results were evaluated based on the presence/absence of the inhibition ring, followed by its diameter determination. Bacterial sensibility to antimicrobial agent is directly proportional to this latter parameter [36].

#### 2.2.6. Cytotoxic Activity towards Hepatocellular Carcinoma (HepG2) Cells

##### HepG2 Seeding on Scaffolds

Seven layered, 20 mm-diameter scaffolds made with ALG, ALG/CNC or ALG/CNC-AgNP were produced and sterilized as reported above. AgNPs were used at different concentrations, ranging from 100 to 250 ppm. The scaffolds were placed in 12-well plates and washed with ultrapure sterile water to eliminate the ethanol. HepG2 were then seeded onto scaffolds or on the nude plate (control cells) at the concentration of 1.3 × 10^6^ cells/well; cells were maintained in Dulbecco’s Modified Eagle’s Medium (DMEM) with 10% fetal calf serum (FCS). In particular, the seeding procedure implied the deposition of 150 μL of cell suspension (containing the 1.3 × 10^6^ cells), to let cells to interact with the scaffold, and after one hour further 850 μL of cell-free medium were added to reach 1 mL of total volume. Plates were kept in an incubator at 37 °C and 5% of CO_2_. The experiment was replicated over 7 days, setting as sampling time points day 2 and 7.

To evaluate the cytotoxicity of the AgNP only, HepG2 cells were seeded on the 12-well plates and exposed to a solution of 100 or 200 ppm AgNP. The nanoparticles were, sterilized by filtration through a 0.45 μm filter before addition to the cells. This experiment was conducted over 48 h, taking into consideration 24 h and 48 h as sampling time points.

##### Viability MTT Assay

AgNP cytotoxicity on HepG2 cells was indirectly evaluated through MTT viability assay.

At each time point, 200 µL of MTT (1mg/mL)-enriched DMEM at 5%FCS were added to each well and incubated for two hours at 37 °C under a continuous flow of 5% CO_2_ in the dark. The scaffolds were then transferred into clear plates. The formazan crystals were dissolved in 500 µL/well DMSO under shaking for 10 min. An aliquot of 150 µL was thus taken and absorbance was finally measured at a wavelength of 570 nm using a Spark^TM^ 10M multimode microplate reader. The absorbance value of only DMSO and scaffold alone was subtracted. Viability percentages were obtained keeping ALG absorbance values (positive control) minus the absorbance of the negative controls as the 100% of viability.

#### 2.2.7. Cells Collection for Proteomic Analysis

Cells grew on a nude plate (control^−^) were washed once in phosphate buffered saline (PBS 1X), then, trypsin solution was added (500 μL/well) and plates incubated for 5 min; finally, the reaction was stopped by addition of fresh medium (DMDM + FCS) and cells were collected in 1.5 mL vials after centrifugation. Cultured scaffolds were transferred in a new sterile plate, then, 3 mL/well of sodium citrate (55 mM) and EDTA (50 mM) at pH 7.4 were added under orbital shaking for 15 min to dissolve the calcium alginate-based scaffolds to remove cells entrapped in it. Once terminated, the content of each well was collected and centrifuged to separate cells from the supernatant (stored at −80 °C); the pellet was then resuspended in 3 mL of PBS to wash cells, centrifuged, the supernatant was eliminated and cells were stored at −80 °C.

Cell culture medium was removed from each culture well designated to proteomic analysis and stored in 1.5 mL Eppendorf at −80 °C until use.

#### 2.2.8. Protein Extraction and Sample Preparation

Total proteins were extracted from cell pellets using 8M urea lysis buffer in Tris-HCl pH 8. Lysates were sonicated for 10 min at 4 °C and incubated for 45 min on ice. Extracts were centrifuged at 4 °C for 10 min at 15,000 rpm and then assayed for protein dosage according to Bradford’s method. Buffer exchange and protein concentration was performed using 50 mM ammonium bicarbonate in Amicon Ultra 0.5 mL centrifugal filters (Sigma-Aldrich) (Darmstadt, Germany) with a 3-kDa MWCO. Then proteins were reduced and alkylated with DTT (5 mM for 30 min at 37 °C), followed by IAA (14 mM for 30 min in the dark), and addition of DTT (5 mM for 15 min at room temperature). Finally, trypsin in 1:50 (enzyme:protein) ratio was used overnight at 37 °C to digest proteins.

#### 2.2.9. Liquid Chromatography-Tandem Mass Spectrometry (LC-MS/MS) Analysis

The resulted tryptic peptides were reconstituted in 50 µL of 0.1% (*v/v*) formic acid aqueous solution and 50% (*v/v*) acetonitrile and analyzed by a liquid chromatography-tandem mass spectrometry (LC-MS/MS) selected reaction monitoring (SRM) approach to identify and quantify proliferative and apoptosis marker proteins. Samples were analyzed as biological (n = 3) and technical replicates (n = 3) using an Agilent HP 1260 liquid chromatography (Agilent Technology, Santa Clara, CA, USA) equipped with a 200-vial capacity sample tray and coupled to a QTRAP 400 triple quadrupole mass spectrometer (ABSCIEX), interfaced with an electrospray source (ESI). An Aeris C18 (150 × 2.1 mm, 5 µm) column (Phenomenex, Torrance, Ca, USA) was used for peptide separation. The injection volume was 10 µL. An elution system based on a solvent gradient [solution A: 0.1% aqueous formic acid (*v/v*)/solution B: 0.08% formic acid in acetonitrile (*v/v*)] was delivered at 0.2 mL/min. A flow rate of 200 µL/min and a 120 min linear gradient was applied for peptide separation.

Source parameters were set as follows: ESI voltage 5.5 kV, declustering potential 70 eV, ion source temperature of 350 °C, collision energy (CE) 40 V. The sheath gas (nitrogen, 99.999% purity) and the auxiliary gas (nitrogen, 99.998% purity) were delivered at flow-rates of 45 and 5 arbitrary units, respectively. As for quantitative analysis, experiments were performed under positive ion-SRM conditions using nitrogen as collision gas (medium nitrogen pressure) and a 20 ms-dual time for each transition monitored. The SRM transitions monitored were reported on Table S1 (Supplementary Material). The analytes were relatively quantified among samples by normalization with total protein content.

#### 2.2.10. Software for Bioinformatic Analysis and Mass Spectrometry (MS) Data Processing

A panel of 6 proliferative and apoptotic proteins (ki-67, HGF, BCLX, Casp8, Casp9 and Casp3) were considered for the assay. FASTA protein sequences was obtained from Uniprot Human proteome database and SRM transitions for each protein were simulated by Skyline (v. 20.0, SCIEX) (Redwood City, CA, USA), setting trypsin as digestion mode with no missed cleavage and carbamidomethylation of cysteins as structural modification (Table S1). Uniqueness of candidate peptide sequences were assessed by BLASTp tool (basic local alignment search tool; www.ncbi.nml.nih.gov link NCBI BLAST) search (algorithm: blastp; ATRIX PA 30; GAP COASTS: existence 10, extension 1; DATABASE: non redundant protein sequences) from NCBI (National Center for Biotechnology Information) (Bethesda, MA, USA).

#### 2.2.11. Statistical Analysis

Data of MTT assay were analyzed by ANOVA one way using the Prism statistical analysis package version 6 (GraphPad Software). Data are given as mean ± standard deviation (SD). A value of *p* < 0.05 was considered statistically significant.

## 3. Results and Discussion

### 3.1. Three-Dimensional (3D) Scaffold Preparation and Characterization

Based on the definition of the optimal parameters for the development of polymer ink [24], ALG and CNC were mixed in different ratio in order to obtain an homogeneous solution with viscosity suitable for the printing of scaffolds. When CNC was mixed in the 3 or 4% *w/v* range with a 5% *w/v* ALG solution, scaffolds with controlled and reproducible 3D structural quality were obtained. Higher percentage of CNC (>5%, *w/v*) resulted in too viscous solutions and lowest percentage of CNC (<3%, *w/v*) presented an high percentage of free water that induced a remarkable swelling of the material during the freezing process. As previously reported, this effect can reduce both resolution of the printing process and of the mechanical strength of the material [31].

The water amount in the hydrogel system can represent a predictive value in terms of compatibility with a living host tissue, since human body is composed of water for its majority, a significantly different hydrated polymeric system could provoke water and osmotic imbalance [37,38]. Moreover, maximum water amount in the system gives indications about the wettability of the medication, another crucial requirement for a hydrogel designed to be capable of absorbing large amounts of exudates while keeping the wound moist.

Measured water content of the scaffolds obtained by using ALG only resulted to be 93.18 ± 0.88% (n = 5), whereas the scaffolds containing ALG and 3% or 4% *w/v* of CNC resulted in being not significantly different (*p* > 0.05) being 86.24 ± 0.45%, 87.12 ± 0.64% (n = 5), respectively.

**Figure 1:**
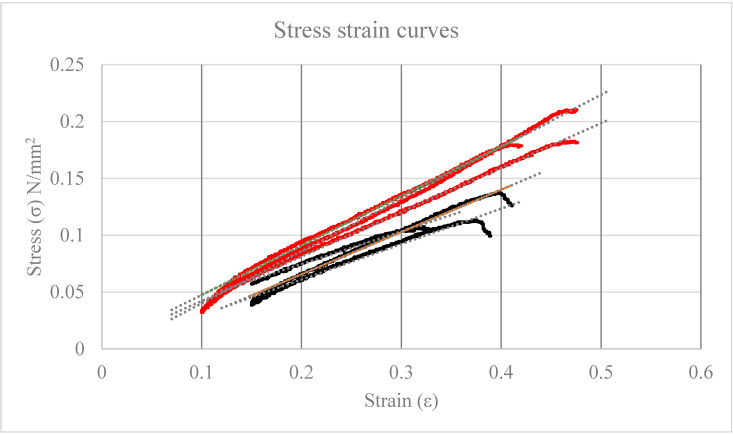
Relevant stress-strain curves: ALG controls (Y = 0.32X − 0.0045, r^2^ = 0.989 n = 3) in RED; alginate/nanocrystalline cellulose (ALG/CNC, 5% and 3% *w/v*) + silver nanoparticles (AgNP, Y = 0.43X − 0.0041, r^2^ = 0.998 n = 3) in black.

Elasticity of hydrated scaffolds was determined by traction tests and subsequently elaborated stress-strain curves (Figure 1).

ALG/CNC and ALG based scaffolds resulted being resistant to breakage till the application of 3.58 ± 0.44 and 5.4 ± 0.53 N, respectively. Elasticity resulted significantly higher (*p* = 0.031) in CNC-containing scaffolds (average *E* resulted 0.32 ± 0.04 MPa) with respect to ALG-based scaffold (0.43 ± 0.03 MPa). No significant differences were observed by using CNC at the 3% or 4% (*w/v*).

Optimal elasticity values for the purpose are not trivial to be defined, these results suggest that the 3D ALG/CNC based scaffolds could be applicable as covering advanced dressing. All the subsequent work was carried out by using the scaffolds prepared with 3% (*w/v*) CNC.

### 3.2. Scanning Electron Microscopy (SEM) and Energy-Dispersive X-ray Spectroscopy (EDS)

**Figure 2:**
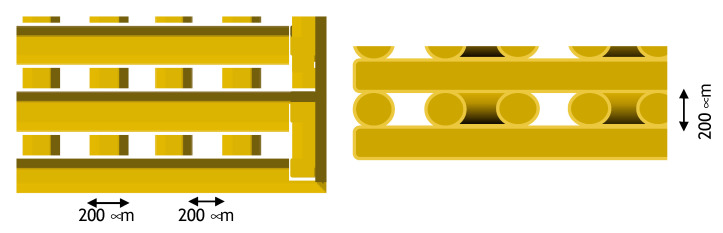
Schematic representation of expected scaffold micro-dimensions.

CAD design for the 3D printing of scaffolds with the nominal filament and macropore values is presented in Figure 2.

**Figure 3:**
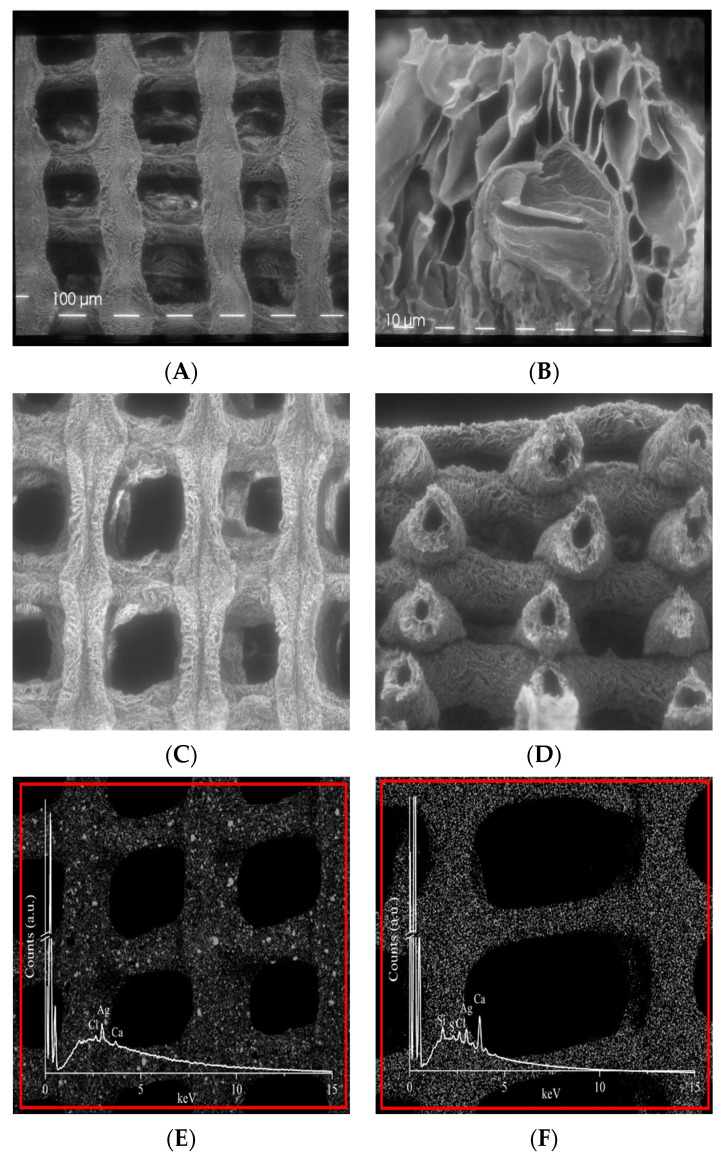
Scanning electron microscope images at different magnification of: (**A**) ALG 5% (*w/v*) scaffold (80×); (**B**) ALG 5% (*w/v*) scaffold (360×); (**C**) ALG/CNC (5% and 3% *w/v*) scaffold (80×); (**D**) ALG/CNC (5% and 3% *w/v*) scaffold cross section (80×). Energy-dispersive X-ray spectroscopy (EDS) map distribution and EDS spectrum of Ag in: (**E**) ALG 5% (*w/v*) scaffold (80×); (**F**) ALG/CNC (5% and 3% *w/v*) scaffold (80×).

As for morphology, SEM images of ALG and ALG/CNC scaffolds exhibited rough homogeneous surfaces with optimal macro pore size, ranging from 174 to 201 μm, demonstrating that the computational CAD parameters set during the design phase are predictable and respected over the whole manufacturing process (Figure 3A,C).

The cross-sectional micrographs of ALG scaffolds revealed a regular interconnected and layered pore structure (Feret diameter ranging from 11 to 45 μm) in the interior region (Figure 3B). Interestingly, the case of ALG/CNC scaffold the regular formation of hollow tubular fibers (Feret diameter ranging from 11 to 126 μm) was observed (Figure 3D). These interesting results can be related only to the biomaterial blend and the gelation parameter process and can be further investigated as a function of the CNC ratio and the processing parameters to create ad hoc 3D systems suitable for applications in regenerative medicine or drug delivery. These porosity values indicate that the topography of the system is suitable for capillary absorption of exudate, wound transpiration and nutrients diffusion through the structure coming from surrounding healthy tissue.

Finally, EDS Ag map clearly evidenced how AgNPs are homogeneously embedded and distributed in both biomaterials (Figure 3E,F).

### 3.3. Antimicrobial Activity Assay

In order to evaluate the antimicrobial efficacy of the developed devices, ALG, ALG/CNC (AgNP-free) and ALG/CNC + AgNP 3D scaffolds were compared in terms of inhibition of bacterial growth in bacterial inoculated Petri dishes. Different AgNP concentrations were tested to assess MIC, when embedded in the 3D scaffolds and relevant data are reported in Table 1. As expected, antimicrobial assay demonstrated how controls (tested scaffolds not containing AgNP) did not show any antimicrobial activity whereas AgNP-loaded scaffolds demonstrated the capability to inhibit growth of both the resistant bacteria species, inducing an inhibition ring of 6 mm. By decreasing concentrations of AgNP from 100 to 1 μg/mL, MIC was established being very close to 10 μg/mL.

By applying scaffolds up to three times, analogous results were obtained. These findings suggest that the ALG/CNC hydrogel formulation can be effective at low dose of AgNPs, with a possible controlled release of the active agent driven by the biomaterial, thus reducing therapeutic side effects and preparation costs.

### 3.4. Cytotoxic Activity Towards HepG2 Cells

#### 3.4.1. Cell Viability Evaluation

As a last application, HepG2, epithelial adherent hepato-carcinoma cells, were used as the cancer model. This cell line is particularly useful for investigating intracellular trafficking, estimating toxicity of xenobiotics or drugs and testing of novel anti-cancer active principles.

Initially, aqueous nanoparticle suspension at 100 and 200 ppm were tested to explore which AgNP concentration could be appropriate for scaffold toxicity experiment.

**Figure 4:**
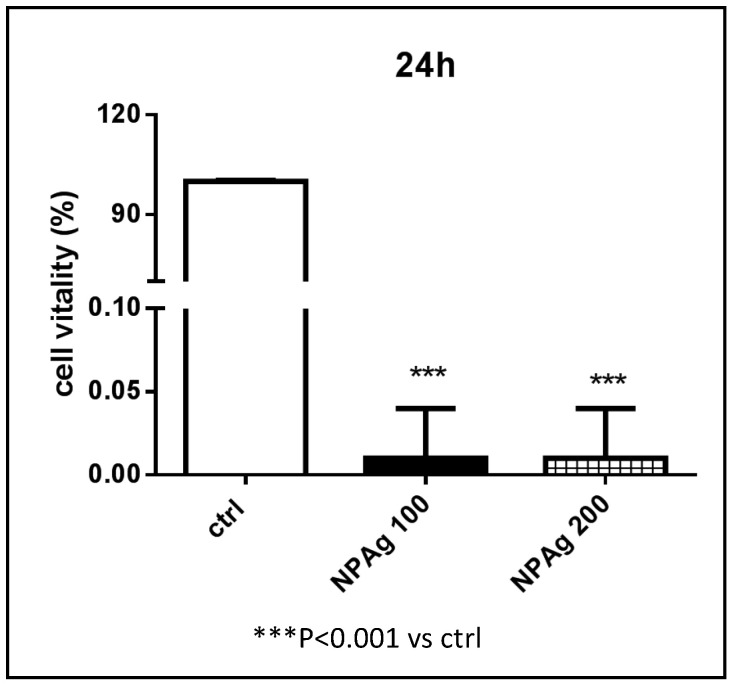
Cell viability % of hepatocellular carcinoma (HepG2) cells NOT TREATED (ctrl), TREATED with AgNP suspension at 100 (NPAg 100) and 200 (NPAg 200) ppm.

Cytotoxicity effect provided by AgNP at both concentration levels results in being clearly high and fast when the suspension is directly administered to cells. Cells vitality immediately drops close to zero over 24 h (Figure 4) and is maintained until 48 h (data not shown).

These findings suggested the use of the lowest AgNP dose (100 ppm) to develop a 3D scaffold presenting cytotoxic activity vs. HepG2 cells meanwhile lowering potential side effects.

**Figure 5:**
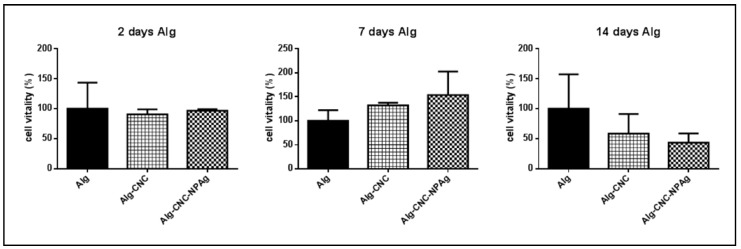
Cell viability % of HepG2 cells on ALG (5% *w/v*), ALG/CNC (5% and 3% *w/v*) and ALG/CNC (5% and 3% *w/v*) + AgNP (100 ppm) scaffolds.

However, ALG/CNC+ AgNP (100 ppm) scaffolds did not exhibit any cytotoxic effect and HepG2 cells’ vitality was comparable to that obtained by using ALG and ALG-CNC control scaffolds within 7 days (Figure 5).

After 14 days the vitality of HepG2 cells significantly decreased on scaffolds containing AgNPs (100 ppm). Interestingly, running this experiment it was noticed that ALG scaffold free of CNC tended to partially deform and/or dissolve while CNC-including scaffolds did not change even under optical microscopy evaluation (images not shown). This can be due to the formation of a tight network of hydrogen bonds between CNC particles and the alginate matrix, that can contribute to a major stability of the scaffold.

The third experiment, AgNP concentration was increased to 250 ppm to better investigate the phenomenon that previously occurred.

**Figure 6:**
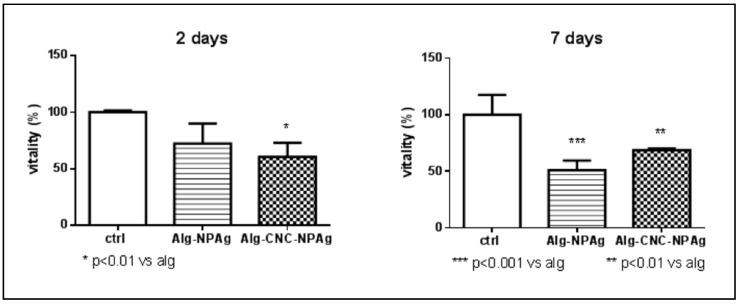
Cell viability % of HepG2 cells NOT TREATED (ctrl), TREATED with ALG (5% *w/v*)-AgNP (250 ppm) and ALG/CNC (5% and 3% *w/v*) + AgNP (250 ppm) scaffolds.

Analysis performed at day 2 revealed that treatments with AgNPs embedded in both ALG and ALG/CNC scaffolds induced a significant reduction of cell viability. In particular, the decrease was statistically significant (*p* < 0.01) in the case of ALG/CNC + AgNP (decrease > 40%) (Figure 6).

At day 7, both scaffolds still showed significant cytotoxicity raise compared to control (*p* < 0.001 and *p* < 0.01, respectively). Cytotoxicity of ALG + AgNP scaffolds showed an increasing pattern over 7 days, making us hypothesize that AgNP concentration in the well solution is increased by being released from alginate melting, and thus having a stronger effect. As already, noticed ALG + AgNP scaffolds lose their structure and dissolve in the presence of HepG2 cells.

These observations suggest the efficacy of the developed scaffold to perform cytotoxic effects and the possibility to test different ALG/CNC ratio in order to develop an erodible system capable of cytotoxic effects towards HepG2 cells in a controlled way over time.

#### 3.4.2. Evaluation of Apoptosis Induction through LC-MS/MS Protein Determination

Several studies available in the literature reported that AgNPs enhance programmed cell death mechanisms. In an endeavour to further investigate the complex process of AgNP cytotoxic/activity on HepG2 cells, specific proteins were determined by LC-MS/MS SRM targeted analysis. In particular, pro-apoptotic initiation caspases (8 and 9), executioner caspases (3), anti-apoptotic Bcl-xL class proteins, proliferative proteins like Ki-67 and the growth factor HGF (hepatocyte growth factor) were selected.

The presence/absence of such proteins and their relative quantitation was carried out by analyzing cells after 2 days and 7 days of culture on ALG (control), ALG + AgNPs (250 ppm) and ALG/CNC + AgNPs (250 ppm). Protein quantitation by means of Bradford assay was employed to estimate protein quantity in each sample and to normalize data (Table S2).

**Figure 7:**
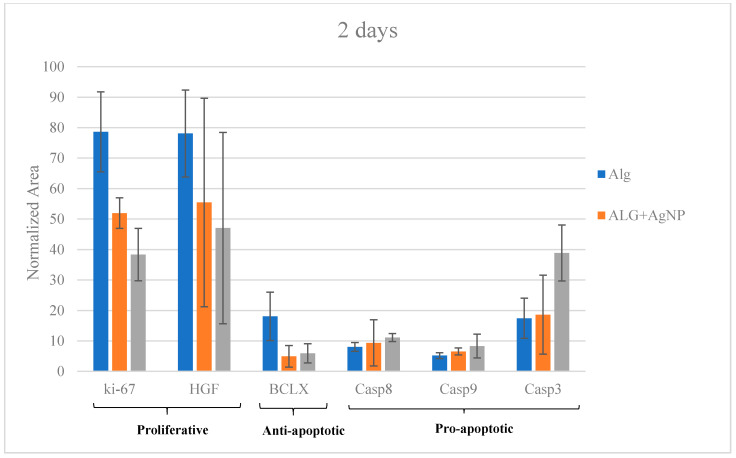
Liquid chromatography-electrospray tandem mass spectrometry (LC-ESI-MS/MS) SRM targeted protein expression at day 2.

At day 2, Ki-67 and HGF are significantly (*p* > 0.05) more expressed in ALG only with respect to both the treating conditions (AgNP-loaded scaffolds) (Figure 7).

**Figure 8:**
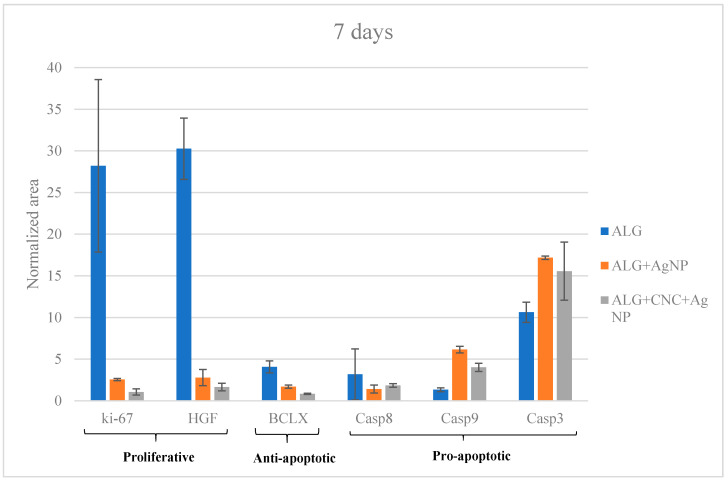
LC-ESI-MS/MS SRM targeted protein expression at day 7.

These outcomes suggested that proliferative activity induced by these two proteins is significantly reduce on the AgNP-containing scaffolds. Confirmation of this hypothesis emerged through the analogue analysis run at day 7 (Figure 8); the expression of Ki-67 and HGF proteins dramatically drop in Ag-NP treated cells (*p* > 0.05) with respect to those cultured on ALG only.

Taking into consideration anti-apoptotic BcL-xL protein, there is a noticeable decrease of its concentration in AgNP-enriched scaffolds at both 2 and 7 days (Figure 7 and Figure 8). The other target proteins, such as pro-apoptotic one (caspases), increased over time in terms of expression for treated scaffolds, strengthening the thesis of AgNP-mediated apoptosis induction (Figure 7 and Figure 8). These outcomes are partially superimposable with those obtained from viability assays and reveals that cells cultured on ALG/CNC + AgNP expressed the lowest viability scores which could be explained by the verified hypothesis of a direct cell apoptosis induction.

## 4. Conclusions

Silver nanoparticles supported in 3D printed ALG/CNC hydrogels were successfully prepared and characterized. Antimicrobial activity and cytotoxicity were evaluated for the first time. The results of an antimicrobial test against S. Aureus and P. aeruginosa confirmed a considerable antimicrobial activity against both Gram+ and Gram− bacteria with low nanoparticle dose (MIC 10 μg/mL) embedded in the composite biomaterial.

In the presence of HEPG2 cells, the data pinpointed that AgNPs embedded in ALG only are rapidly dispersed in the culture medium because of low mechanical properties and solubility. The use of CNC in the ALG/AgNP mixture allowed us to overcome this issue and to obtain scaffolds with improved mechanical properties against HEPG2 cells, making AgNP cytotoxicity more effective over time. Finally, target MS analysis of marker proteins provided evidence about the impact of AgNPs on chosen cellular mechanisms. Hence, the use of this composite can be a promising approach to create new active materials for clinical applications.

## Figures and Tables

**Table 1:** Determination of antimicrobial activity of 3D-printed developed scaffolds.

SCAFFOLD	*Staphylococcus aureus*		*Pseudomonas aeruginosa*	
	Ø Inhibition Diameter (mm)			
ALG	0	0	0	0
ALG/CNC	0	0	0	0
ALG/CNC + AgNP (100 μg/mL)	6	6	6	6
ALG/CNC + AgNP (10 μg/mL)	6	6	6	6
ALG/CNC + AgNP (5 μg/mL)	0	0	0	0
ALG/CNC + AgNP (1 μg/mL)	0	0	0	0

## Supplementary Materials

The following are available online at https://www.mdpi.com/2079-4991/10/5/844/s1: Table S1: List of peptides and SRM transitions monitored, Table S2: Bradford assay protein quantitation (n = 2).

## References

[1] Edwards R., Harding K.G. (2004). Bacteria and wound healing. Curr. Opin. Infect. Dis..

[2] Scott Van Epps J., Younger J.G. (2016). Implantable Device Related Infection. Shock.

[3] Misic A.M., Gardner S.E., Grice E.A. (2014). The wound microbiome: Modern approaches to examining the role of microorganisms in impaired chronic wound healing. Adv. Wound Care.

[4] Serra R., Grande R., Butrico L., Rossi A., Settimio U.F., Caroleo B., Amato B., Gallelli L., De Franciscis S. (2013). Chronic wound infections: The role of Pseudomonas aeruginosa and Staphylococcus aureus. J. Expert Rev. Anti-Infect. Ther..

[5] Hartemann-Heurtiera A., Senneville E. (2008). Diabetic foot osteomyelitis. Diabetes Metab..

[6] Franks L.A., Slansky J.E. (2012). Multiple Associations between A Broad Spectrum of Autoimmune Diseases, Chronic Inflammatory Diseases and Cancer. Anticancer Res..

[7] Percival S.L., Bowler P.G., Dolman J. (2007). Antimicrobial activity of silver-containing dressings on wound microorganisms using an in vitro biofilm model. Int. Wound J..

[8] Jones A., Vaughan D. (2005). Hydrogel dressings in the management of a variety of wound types: A review. J. Orthop. Nurs..

[9] Axpe E., Oyen M.L. (2016). Applications of Alginate-Based Bioinks in 3D Bioprinting. Int. J. Mol. Sci..

[10] Hunta N.C., Smith A.M., Gbureck U., Shelton R.M., Grover L.M. (2010). Encapsulation of fibroblasts causes accelerated alginate hydrogel degradation. Acta Biomater..

[11] Dhivya S., Vijaya Padma V., Santhini E. (2015). Wound dressings—A review. Biomedicine.

[12] Aderibigbe B.A., Buyana B. (2018). Alginate in Wound Dressings. Pharmaceutics.

[13] Knill C.J., Kennedy J.F., Mistry J., Miraftab M., Smart G., Groocock M.R., Williams H.J. (2004). Alginate fibres modified with unhydrolysed and hydrolysed chitosans for wound dressings. Carbohydr. Polym..

[14] Mondal S. (2017). Preparation, properties and applications of nanocellulosic materials. Carbohydr. Polym..

[15] Jorfi M., Foster E.J. (2015). Recent advances in nanocellulose for biomedical applications. J. Appl. Polym. Sci..

[16] Poonguzhali R., Basha S.K., Kumari V.S. (2017). Synthesis and characterization of chitosan-PVP-nanocellulose composites for in-vitro wound dressing application. Int. J. Biol. Macromol..

[17] Ma X., Li R., Zhao X., Ji Q., Xing Y., Sunarso J., Xia Y. (2017). Biopolymer composite fibres composed of calcium alginate reinforced with nanocrystalline cellulose. Compos. Part A Appl. Sci. Manuf..

[18] De France K.J., Hoare T., Cranston E.D. (2017). Review of hydrogels and aerogels containing nanocellulose. Chem. Mater..

[19] Ahmed A., Boateng J. (2018). Calcium alginate-based antimicrobial film dressings for potential healing of infected foot ulcers. Ther. Deliv..

[20] Inbathamizh L., Ponnu T.M., Mary E.J. (2013). *In vitro* evaluation of antioxidant and anticancer potential of Morinda pubescens synthesized silver nanoparticles. J. Pharm. Res..

[21] Zhang X., Liu Z., Shen W., Gurunathan S. (2016). Silver Nanoparticles: Synthesis, characterization, Properties, Applications, and Therapeutic Approaches. Int. J. Mol. Sci..

[22] Singh A., Dar M.Y., Joshi B., Sharma B., Shrivastava S., Shukla S. (2018). Phytofabrication of Silver nanoparticles: Novel Drug to overcome hepatocellular ailments. Toxicol. Rep..

[23] Rai M.K., Deshmukh S.D., Ingle A.P., Gade A.K. (2012). Silver nanoparticles: The powerful nanoweapon against multidrug-resistant bacteria. J. Appl. Microbiol..

[24] Elviri L., Foresti R., Bergonzi C., Zimetti F., Marchi C., Bianchera A., Bernini F., Silvestri M., Bettini R. (2017). Highly defined 3D printed chitosan scaffolds featuring improved cell growth. Biomed. Mater..

[25] Tiwari A., Tiwari A. (2013). Nanomaterials in Drug Delivery, Imaging, and Tissue Engineering.

[26] Chen C.H., Huang T.S., Wong C.H., Hong C.L., Tsai Y.H., Liang C.C., Lu F.J., Chang W.H. (2009). Synergistic anti-cancer effect of baicalein and silymarin on human hepatoma HepG2 Cells. Food Chem. Toxicol..

[27] Wolf-Yadlin A., Hautaniemi S., Lauffenburger D.A., White F.M. (2007). Multiple reaction monitoring for robust quantitative proteomic analysis of cellular signaling networks. PNAS.

[28] Bergamonti L., Potenza M., Poshtiri A.H., Lorenzi A., Sanangelantoni A.M., Lazzarini L., Lottici P.P., Graiff C. (2020). Ag-functionalized nanocrystalline cellulose for paper preservation and strengthening. Carbohydr. Polym..

[29] Basile R., Bergamonti L., Fernandez F., Graiff C., Haghighi A., Isca C., Lottici P.P., Pizzo B., Predieri G. (2018). Bio-inspired consolidants derived from crystalline nanocellulose for decayed wood. Carbohydr. Polym..

[30] Intini C., Elviri L., Cabral J., Mros S., Bergonzi C., Bianchera A., Flammini L., Govoni P., Barocelli E., Bettini R. (2018). 3D-printed chitosan-based scaffolds: An *in vitro* study of human skin cell growth and an *in-vivo* wound healing evaluation in experimental diabetes in rats. Carbohydr. Polym..

[31] Bergonzi C., Di Natale A., Zimetti F., Marchi C., Bianchera A., Bernini F., Silvestri M., Bettini R., Elviri L. (2019). Study of 3D-printed chitosan scaffold features after different post-printing gelation processes. Sci. Rep..

[32] Bergamonti L., Bergonzi C., Graiff C., Lottici P., Bettini R., Elviri L. (2019). 3D printed chitosan scaffolds: A new TiO2 support for the photocatalytic degradation of amoxicillin in water. Water Res..

[33] Plazinski W. (2011). Molecular basis of calcium binding by polyguluronate chains. Revising the egg-box model. J. Comput. Chem..

[34] Godebo G., Kibru G., Tassew H. (2013). Multidrug-resistant bacterial isolates in infected wounds at Jimma University Specialized Hospital, Ethiopia. Ann. Clin. Microbiol. Antimicrob..

[35] Biemer J.J. (1973). Antimicrobial Susceptibility Testing by the Kirby-Bauer Disc Diffusion Method. Ann. Clin. Lab. Sci..

[36] Stanley B.A., Neverova I., Brown H.A., Van Eyk J.E. (2003). Optimizing protein solubility for two-dimensional gel-electrophoresis analysis of human myocardium. Proteomics.

[37] Qin Y. (2004). Absorption characteristics of alginate wound dressings. J. Appl. Polym. Sci..

[38] Akhtar R., Sherratt M.J., Kennedy Cruickshank J., Derby B. (2011). Characterizing the elastic properties of tissues. Mater. Today.

