# Supplementary material for: Three-Dimensional (3D) Printed Silver Nanoparticles/Alginate/Nanocrystalline Cellulose Hydrogels: Study of the Antimicrobial and Cytotoxicity Efficacy
Source: Nanomaterials (Basel). 2020 Apr 28;10(5):844. doi: 10.3390/nano10050844 (PMC7711489; doi:10.3390/nano10050844)
Supplement: Supplementary file 1 [file nanomaterials-10-00844-s001.pdf]

Table S1. List of peptides and SRM transitions monitored.

Acession n.	Protein Name	Peptide Sequence	Precursor Mz	Prec. Char.	Product Mz	Fragment Ion
Q07817	BCL-X	EVIPMAAVK	479,28	2	616,35	y6
Q07817	BCL-X	EVIPMAAVK	479,28	2	229,12	b2
Q07817	BCL-X	EAGDEFELR	533,25	2	693,36	y5
Q07817	BCL-X	EAGDEFELR	533,25	2	288,20	y2
Q14790	CASP-8	MLEESNLSFLK	655,84	2	937,50	y8
Q14790	CASP-8	MLEESNLSFLK	655,84	2	808,46	y7
Q14790	CASP-9	MLEESNLSFLK	655,84	2	1066,54	y9
Q14790	CASP-8	MLEESNLSFLK	655,84	2	245,13	b2
Q14790	CASP-8	EQDSESQTLDK	640,29	2	691,36	y6
Q14790	CASP-8	EQDSESQTLDK	640,29	2	476,27	y4
P55211	CASP-9	LFFIQAC[+57]GGEQK	699,35	2	877,38	y8
P55211	CASP-9	LFFIQAC[+57]GGEQK	699,35	2	408,23	b3
P55211	CASP-9	ELFRPHMIEDIQR	421,72	4	303,18	y2
P55211	CASP-9	ELFRPHMIEDIQR	421,72	4	634,81	b10
P55211	CASP-9	FSSLHFMVEVK	441,90	3	246,18	y2
P55211	CASP-9	FSSLHFMVEVK	441,90	3	545,29	y9
P42574	CASP-3	SGTDVDAANLR	559,78	2	873,44	y8
P42574	CASP-3	SGTDVDAANLR	559,78	2	758,42	y7
P42574	CASP-3	EEIVELMR	509,77	2	647,35	y5
P42575	CASP-3	EEIVELMR	509,77	2	548,29	y4
P42578	CASP-3	GTELDC[+57]GIETDSGVDDDMAC[+57]HK	808,99	3	1012,89	y18
P42579	CASP-3	GTELDC[+57]GIETDSGVDDDMAC[+57]HK	808,99	3	876,33	y7
P46013	ki-67 b	ADVEEEFLALR	646,33	2	1006,52	y8
P46013	ki-67 b	ADVEEEFLALR	646,33	2	877,48	y7
P46013	ki-67 b	VEDAADSATKPENLSSK	587,96	3	767,37	y15
P46013	ki-67 b	VEDAADSATKPENLSSK	587,96	3	229,12	b2
P46013	ki-67 b	VEDAADSATKPENLSSK	587,96	3	201,09	b6
P46013	ki-67 b	VEDAADSATKPENLSSK	851,93	3	831,89	y16
P46013	ki-67 b	SQPDPVDTPTSSKPQSK	600,30	3	792,40	y15
P46013	ki-67 b	SQPDPVDTPTSSKPQSK	600,30	3	216,10	b2
P14210	HGF	GEEGGPWC[+57]FTSNPEVR	911,40	2	701,36	y6
P14210	HGF	GEEGGPWC[+57]FTSNPEVR	911,40	2	802,41	y7
P14210	HGF	HIFWEPDASK	615,30	2	251,15	b2
P14210	HGF	HIFWEPDASK	615,30	2	517,26	y5

Table S2. Bradford assay protein quantitation (n=2).

Treatment	2 Days	7 Days
	extracted proteins (µg)	
ALG	146.5	439.3
ALG	152.0	442.1
ALG+AgNPs (250ppm)	158.6	263.1
ALG+AgNPs (250ppm)	487.6	244.4
ALG/CNC+AgNPs (250ppm)	164.8	288.8
ALG/CNC+AgNPs (250ppm)	140.5	328.0
